# Meta-analysis of the best exercise mode and dose study for improving spinal health

**DOI:** 10.3389/fspor.2025.1614906

**Published:** 2025-09-03

**Authors:** Ziyang Li, Zihan Bao, Shun Wang, Mengqi Zhao

**Affiliations:** College of Physical Education, Huaibei Normal University, Huaibei, Anhui, China

**Keywords:** spinal health, scoliosis, sports rehabilitation, exercise therapy, physical therapy

## Abstract

**Objective:**

To explore the influence of different exercise methods on spinal health through Meta-analysis, and then provide scientific exercise suggestions for different groups of people.

**Methods:**

Randomized controlled experimental studies (RCTs) of different exercise modes on spinal health in CNKI, Wanfang, Web of Science, PubMed, and Ebsco databases were searched. The search dates were limited to self-built databases until February 2025. After screening, 30 articles and 2,105 subjects were included and analyzed using Review Manager 5.4 and Stata 18 software.

**Results:**

Exercise therapy was significantly superior to the control group in relieving pain (SMD = −0.87, 95% CI: −1.11, −0.63), improving cervical spine dysfunction (SMD = −0.90, 95% CI: −1.47, −0.34), improving lumbar spine dysfunction (SMD = −0.59, 95% CI: −0.77, −0.41) and correcting scoliosis (SMD = −0.82, 95% CI: −1.30, −0.34). Through subgroup analysis of heterogeneous sources from seven aspects: disease type, intervention site, intervention mode, intervention time, intervention frequency, intervention period and age,the results show that exercise intervention can effectively improve the pain level and dysfunction in patients with spinal diseases, and help to promote the recovery of Cobb's angle in patients. Subgroup analysis showed that each intervention of 10–30 min, 3–4 times a week and continuous exercise for 10–20 weeks was an ideal exercise plan.

**Conclusion:**

Exercise intervention can significantly improve the health of spinal patients, and the 18-style of Zong Jianji has strong universality and is suitable for the intervention of various spinal diseases. The ideal exercise program is 10–30 min each intervention, 3–4 times a week, and continuous exercise for 10–20 weeks. Baduanjin has better curative effect on patients with low back pain and scoliosis. Each intervention lasts for 30–50 min, exercises 3 times a week or 1–2 times a day, and continuous exercise for 5–6 weeks or 10 weeks is the most practical. In the future, more high-quality, multi-disease clinical studies and evidence should be collected to verify this conclusion.

**Systematic Review Registration:**

https://www.crd.york.ac.uk/PROSPERO/, identifier [CRD420251008053].

## 1 Introduction

Modern lifestyle and living habits have led to increasingly prominent spinal health problems, and the incidence of cervical and lumbar spondylosis is increasing year by year and showing a younger trend. In 2017, among the life losses caused by various non-fatal diseases in China, neck pain surpassed depression and rose to the first place. According to Baidu Health Medical Code Platform, sleep disorder, lumbar disc herniation and cervical spondylosis have become the three major occupational diseases that most trouble the public. Patients with cervical spondylosis aged 20–40 account for 64%. According to the “China Degenerative Spinal Health Report 2023”, cervical and lumbar spondylosis has become one of the leading diseases that cause the loss of healthy life among Chinese people. The incidence of spondylosis in adults is 30%, and the incidence of lumbar spondylosis is 80%. According to the data of orthopedic database platform of Peking University Third Hospital, the proportion of cervical spine surgery among people aged 35–49 is second only to those aged 50–64, and the proportion of lumbar spine surgery under 35 has also increased significantly in recent years. Spinal and lumbar spondylosis is no longer a geriatric disease (1, 2). The spine serves as the core pillar of the human body, supporting the upper body and protecting the spinal nerves, and its mechanical balance is essential for systemic function (3). Abnormal spine will lead to mechanical imbalance between joints, and the tissues around skeletal muscle will receive great distortion force, which can easily lead to a series of diseases such as facial asymmetry, fatigue of muscles, nerves and fascia, and decreased cardiopulmonary function (4). Teenagers are prone to scoliosis because they work at their desks for a long time, ranging from bad posture to affecting cardiopulmonary function. Sedentary people in the workplace can easily lead to cervical and lumbar diseases, and the elderly have a high incidence of spinal stenosis and other diseases due to degenerative diseases. In order to cope with the systemic chain reaction such as nerve compression, decreased cardiopulmonary function and dyskinesia caused by spinal imbalance, it is necessary to strengthen early intervention and daily protection (5–7).

Based on population survey data, common relief measures for lumbar and cervical spine discomfort include shoulder and neck relaxation exercises, physical massage, topical drugs and office aids. As a non-invasive intervention method, exercise intervention has been widely verified to promote spinal health. A large number of studies have shown that reasonable exercise can enhance the muscle strength around the spine, provide better support for the spine, disperse the pressure on the spine, and improve the stability of the spine (8). At the same time, exercise can also promote the blood circulation of the spine, provide sufficient nutrients for spinal tissues, help repair damaged tissues and slow down the degeneration process of the spine. However, there are differences in the mechanism of action and effects of different exercise modes on the spine (9, 10) It is not known which exercise mode intervention works best, such as poorly selected exercise mode or excessive exercise intensity, which may also cause damage to the spine. In addition, in the past, Meta-analysis mostly focused on the intervention effect of a single exercise mode on a single disease type, and the conclusions drawn by each study were different (8–11). Based on this, this paper explores the impact of different exercise methods on spinal health through Meta-analysis, in an attempt to find an exercise method that can effectively intervene in various spinal diseases, customize scientific exercise programs for different groups of people, and achieve the goal of “Healthy China 2030” 79-year-old life expectancy by optimizing the effectiveness of spinal health management. This health promotion model with exercise intervention as the core not only conforms to the development direction of “integration of physical health” in the Outline of Building a Sports Power, but also provides a replicable innovative path for the prevention and control of chronic diseases. It is becoming a practical example of deep integration of national fitness and national health in the new era.

## 2 Data and methods

### 2.1 Literature search strategy

#### 2.1.1 Database and retrieval time

The article follows the requirements of “There are Priority Reporting Projects for Systematic Reviews and Meta-analyses” (PRISMA) to conduct Meta-analysis and complete protocol registration (registration number: CRD420251008053) on the PROSPERO platform to improve research transparency and objectivity (12). Relevant literature was obtained through CNKI, Wanfang, Pubmed, Web of Science and Ebsco databases. The search time limit was from the establishment of each database to February 2025. The first and second authors used an independent double-blind method to search the literature.

#### 2.1.2 Search strategy

In order to explore the effects of different exercise modes on spinal health, according to PRISMA guidelines, searches were conducted from Pubmed, CNKI, WanFang Data, and WOS databases. The search period was set from the establishment of the database to April 2025. This study was conducted from April to June 2025, and was continuously updated according to newly published research results. The search query was: (Exercise spine strengthening, OR spine OR cervical spine OR lumbar spine OR thoracic spine OR sacral spine OR caudal spine OR neck-shoulder syndrome OR scoliosis OR chronic non-specific low back pain OR mandatory spondylitis OR non-specific low back pain OR upper cross syndrome OR Lumbar disc herniation) AND (yi zong's 18 styles of making the spine healthy OR Baduanjin OR Tai Chi OR Yoga OR Pulling Exercises OR Resistance Exercises OR Pilates OR Core stability training OR Schrotter Therapy OR Exercise Therapy OR Core stability training OR Schrotter Therapy OR In order to ensure the accuracy of data extraction, two independent researchers were arranged to carefully review the key information such as the title and abstract of the article, screen out the studies that met the requirements, and extract the following information: the author of the literature, the year of publication, the gender and age distribution of the subjects, the total sample size, the specific intervention means, the detailed dose of the intervention (covering frequency, period and duration), and the final outcome indicators. For any disagreement, a group discussion with the third investigator will be held to determine the final literature to be included. After the literature search was completed, the literature was imported into Endnote for deduplication, and the literature was independently screened by two researchers.

### 2.2 Literature inclusion and exclusion criteria

According to the PICOS principle, two researchers screened the literature from five aspects: Patients, Intervention, Comparisons, Outcomes and Study Design. The results showed that there were differences in opinions. Resolved through discussion or ruled by a third party.

P (Population): The subjects of the study were people with spinal diseases.
I (Intervention measures): The intervention measures are exercise therapy such as Yizong Jianji Eighteen Poses, Baduanjin, Tai Chi Chuan, yoga, traction exercise, resistance exercise, Pilates, massage, core training and Schroter.
C (control): Comparison between participants who underwent exercise intervention and those who did not during the experiment.
O (outcome measures): ① Visual Analogue Scale (VAS) ② Cervical spine dysfunction scale (NDI). ③ Oswestry dysfunction index (ODI). ④ Cobb angle.
S (Study Design): The review included raw randomized controlled trials in parallel group or crossover design conducted on humans.

Inclusion criteria: ① The type of included literature is RCT experiment; ② The study subjects are people with spinal diseases; ③ The intervention measures were Zongjianji Eighteen Poses, Baduanjin, Tai Ji Chuan, yoga, pulling exercise, resistance exercise, Pilates, massage, core training and Schroter; ④ Outcome measures: VAS (visual analogue scale), NDI (neck disability index), ODI (Oswestry disability index), Cobb angle.

Exclusion criteria: ① Exclude books, conferences, graduation theses, opinion articles, observational studies, speech abstracts and other types of literature; ② Review, systematic review and conference; ③ Animal experiments or case studies; ④ Repeated publication of literature; ⑤ Data cannot be obtained.

### 2.3 Literature screening

The retrieved literature was imported into Endnote21 software for deduplication, and then the two researchers independently read the title and abstract of the literature according to predetermined criteria, and then decided whether to include it. In case of disagreement, third party intervention was sought. Double-blind independent evaluation is adopted in the full-text review stage, and the dispute handling process is consistent with the initial screening, and the screening quality is ensured by random review and consistency test.

### 2.4 Assessment of risk of literature bias

Two investigators used the revised Cochrane Risk of Bias Instrument for Randomized Trials (RoB2) to evaluate the literature quality of included studies from six dimensions: selectivity bias, implementation bias, measurement bias, follow-up bias, reporting bias, and other biases. The RoB2 was covering the following evaluation domains: bias arising from the randomization process, bias due to deviations from intended interactions, bias due to missing outcome data, bias due to measurement of the outcome and bias in selection of the reported result. Two independent reviewers LZY and BZH assessed the risk of bias, and in cases of disagreement, a third reviewer WS was consulted to reach a consensus. This approach ensures the reliability and reproducability of our findings.

### 2.5 Data extraction

The two reviewers independently extracted data from eligible studies, including author, year, participant characteristics, study design, training protocol, exercise test type, and quality of life indicators (M ± SD), and stored them in a preset Excel sheet. A third reviewer cross-checks data integrity and converts measurements from different units with Review Manager if necessary to ensure data consistency.

### 2.6 Outcome measures

The main outcome measures were: ① Visual Analogue Scale (VAS) ② Neck Disability Index (NDI). ③ Oswestry Disability Index (ODI). ④ Cobb angle.

### 2.7 Statistical analysis

Review Manager 5.4 and stata 18 data analysis software were used for synthetic analysis, subgroup analysis and forest plot production, and the subgroup analysis of exercise elements included in the study was performed to explore the effects of different exercise modes on spinal health-related outcome measures. In order to minimize the heterogeneity caused by different units, measurement methods, scoring standards, etc., this paper uses standardized mean difference (SMD) and 95% confidence interval as effect analysis statistics, and the difference is statistically significant when the statistical significance is set at *p* < 0.05. When *p* > 0.05, it is considered that there is no statistically significant difference between the combined two groups. In this study, the effect model was determined according to the heterogeneity *I*^2^ value in the Cochrane Manual of Systematic Reviews. If there was no or small heterogeneity between studies (*I*^2^ ≤ 50%, *p* ≥ 0.1), the fixed effect model was used for pooled analysis; If heterogeneity was significant (*I*^2^ > 50%, *p* < 0.1), pooled analyses were performed using random effects models. When *I*^2^ > 50%, sensitivity analysis was used to assess the stability and reliability of the results. Publication bias was analyzed using Egger's test when at least 10 studies were included in the Meta-analysis (13). In addition, subgroup analysis was performed, and the studies under each outcome measure were grouped from six aspects: intervention site, exercise mode, intervention time, intervention frequency, intervention period and age, and Meta-analysis was performed to detect heterogeneous sources.

## 3 Results

### 3.1 Literature screening process and results

**Figure 1:**
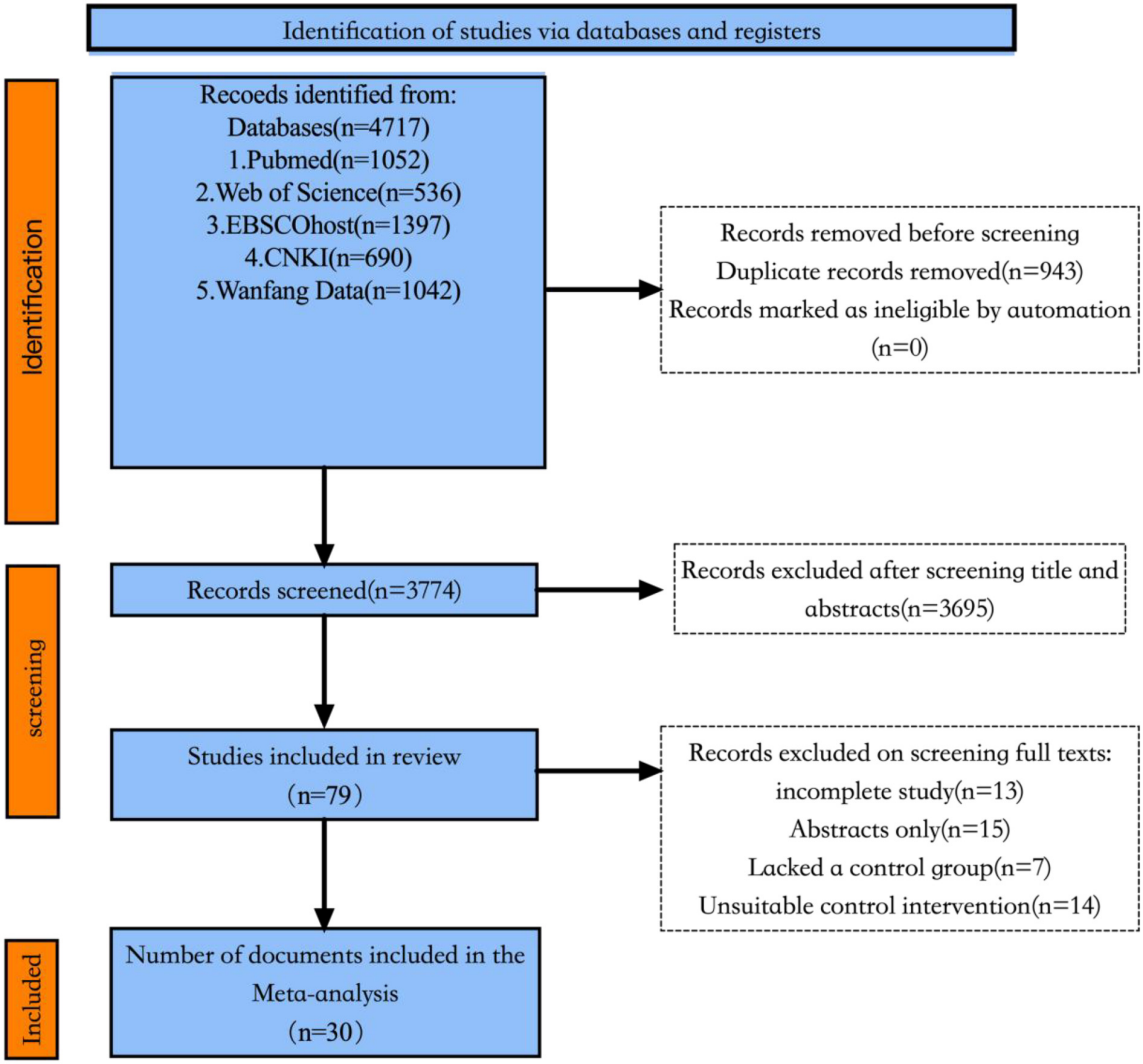
Flow chart of literature screening.

A total of 4,717 articles were obtained through literature search. Subsequently, 943 articles were eliminated due to duplication. After analyzing the titles and abstracts, 3,695 articles were excluded because they did not meet the main criteria and themes of this review. Of the remaining 79 articles, 49 were excluded after reading the full text (15 studies had only abstracts, 7 studies lacked control groups, 13 studies had incomplete data, and 14 studies had unreasonable control measures). Finally, 30 articles were included, of which two articles (14, 15) contained two experimental groups and a control, which can be divided into two groups of controlled studies, that is, a total of 30 articles and 32 RCT studies were included in this study. The literature screening process and results are shown in Figure 1. See Figure 1 for details of the literature screening process.

### 3.2 Characteristics of included studies

A total of 2,105 subjects were included in this study, including 1,062 in the exercise therapy group and 1,043 in the control group. The exercise therapy group was intervened with exercise therapy such as Baduanjin, Zongjianji 18-style, traction exercise and resistance exercise, while the control group was treated with other conventional therapies. The basic characteristics of the included studies are shown in Table 1.

**Table 1:** Basic characteristics of included studies.

Included study literature (First author)	Experimental group			Control group			Time and frequency of intervention	Outcome index	Type of disorder
	Number of	Age/years	Interventions	Number	Age	Interventions			
Chong Yuping 2011 (16)	30	42.66 ± 12.62	Yoga	30	43.28 ± 11.75	N	60 min/time for 6 weeks, once every other day	①	Rehabilitation of non-specific low back pain
Chong Yuping 2014 (17)	30	38.87 ± 10.41	Yoga	30	38.87 ± 10.41	N	60 min/time for 8 weeks, once every other day	①	Cervical spondylosis cervical type
Bao Shanju 2023 (18)	31	–	Tai Ji Chuan	29	–	N	35 min/time for 12 weeks, 3 times/week	①②	Superior crossover syndrome
Ke Xiaojian 2018 (19)	20	19.5 ± 0.6	Baduanjin	17	19.5 ± 0.6	N	3–4 times/time in 10 weeks, ≥ 5 times/week	①②	Neck-shoulder syndrome
Liu Lu 2023 (20)	48	11.52 ± 0.50	Long Yincao	48	11.48 ± 0.50	N	30 min/time for 12 weeks, once every two days	④	Mild idiopathic scoliosis
Li Lin 2021 (21)	60	>20	Yizong strengthens the spine	60	>20	N	20 min/time, 1 time/day	①②④	Cervical spondylosis cervical type
Lin Li 2023 (22)	30	41.39 ± 1.77	Yizong strengthens the spine	30	43.21 ± 1.63	N	10 min/time, 1 time/day	①	Lumbar disc herniation
Hu Xingping 2018 (23)	30	62.80 ± 2.31	Yizong strengthens the spine	30	62.6 ± 2.88	N	12 weeks	①	Lumbar spinal stenosis
Ding Yong 2014 (24)	22	61. 05 ± 4. 66	Baduanjin	18	60. 89 ± 4. 92	N	40 min/time for 12 weeks, 5 times/week	①④	Poor posture of spine
Weimin Zou 2023 (25)	60	21.3 ± 6.3	Resistance exercise	60	21.6 ± 5.9	N	60 min/time for 12 weeks, 3 times/week	①②	Chronic low back pain
Wang Kaile 2024 (26)	18	12.1 ± 1.8	Core Training	15	11.9 ± 2.2	N	50 min/time for 10 weeks, 3 times/week	④	Neck-shoulder syndrome
Song Hua 2008 (27)	37	42.69 ± 15.38	Tai Ji Chuan	31	40.72 ± 13.10	N	60 min/time for 24 weeks, 6 times/week	①	Idiopathic scoliosis
Qiu Peng 2014 (14)	10	—	Pulling motion	10	–	N	15 min/time for 6 weeks, 2 times/week	①	Lumbar disc herniation
	10	–	Swiss ball	10	–	N	15 min/time for 6 weeks, 2 times/week	①	
Zeng Fanling 2020 (28)	40	12–18	Core Training	40	11–17	N	35 min/time for 10 weeks, once/day	④	Idiopathic scoliosis
Li Xiuyuan 2024 (29)	50	42.98 ± 10.21	Baduanjin	50	43.59 ± 10.17	N	12 weeks 25–30 min/time, 2 times/day	①②	Neck-shoulder syndrome
Aboufazeli2021 (30)	12	38.8 ± 5.8	Resistance exercise	12	39 ± 5.9	N	8 weeks, 3 times/week	①③	Chronic low back pain
FAlla2013 (31)	23	39.1 ± 8.7	Resistance exercise	23	38.6 ± 9.0	N	8 weeks 40–50 min/time, 1 time/week	①②	Chronic neck pain
Seyda2016 (32)	51	47 ± 10	Manipulative therapy	51	44 ± 13	N	75–80 min/time for 4 weeks, 3 times/week	②	Neck pain
Lidiane2024 (33)	8	31.3 ± 2.7	Pilates	7	30.8 ± 2.4	N	12 weeks 50 min/time, 2 times/week	③	Back pain
Fortin2023 (34)	25	45.16 ± 10.66	Pulling motion	25	37.6 ± 11.6	N	45 min/time for 12 weeks	③	Low back pain
Erika2014 (35)	21	52.8 ± 10.6	Pulling motion	20	51.3 ± 6.3	N	60 min/time for 10 weeks, 2 times/week	①	Fibromyalgia
Farzaneh2022 (15)	12	42.58 ± 14.18	Pilates in water	14	38.07 ± 8.69	N	6 weeks, 4 times/week	①	Ankylosing spondylitis
	14	39.21 ± 10.25	Water stretch	14	38.07 ± 8.69	N	6 weeks, 4 times/week	①	
Zhou2022 (36)	130	44.36 ± 10.44	Tai Ji Chuan	130	51.77 ± 10.32	N	50 min/time for 6 weeks, 3 times/week	①③	Lumbar disc herniation
Jeremy2024 (37)	15	48.53 ± 18.48	Pulling motion	15	59.13 ± 15.84	N	25 min/time for 5 weeks, 5 times/day	③	Spinal growth and pain
Kyung2022 (38)	16	37.5 ± 10.6	Resistance exercise	16	35.8 ± 8.0	N	40 min/time for 6 weeks, 3 times/week	②	Neck-shoulder syndrome
Guo2025 (39)	37	19 ± 0.97	Pulling motion	37	18.92 ± 0.86	N	45 min/time for 8 weeks, 5 times/week	①②	Superior crossing syndrome
Karina2024 (40)	26	11.6 ± 1.1	Schrotter	17	12.5 ± 1.4	N	2 years	④	Idiopathic scoliosis
Zhang 2024 (41)	31	13.42 ± 1.06	Schrotter	31	13.97 ± 1.21	N	90 min/time for 12 weeks, 3 times/week	④	Idiopathic scoliosis
Lorena2022 (42)	20	34 ± 20.74	Massage	20	43 ± 23.70	N	30 min/time for 5 weeks, 2 times/week	③	Chronic non-specific low back pain
Sheng2020 (43)	30	39.3 ± 9.2	Baduanjin	30	40.6 ± 10.3	N	10 weeks, 2 times/week	③	Discogenic low back pain

“–” means not reported. *N* is routine rehabilitation, ①: VAS; ② NDI; ③ ODI; ④ Cobb.

### 3.3 Bias analysis of included literature

**Figure 2:**
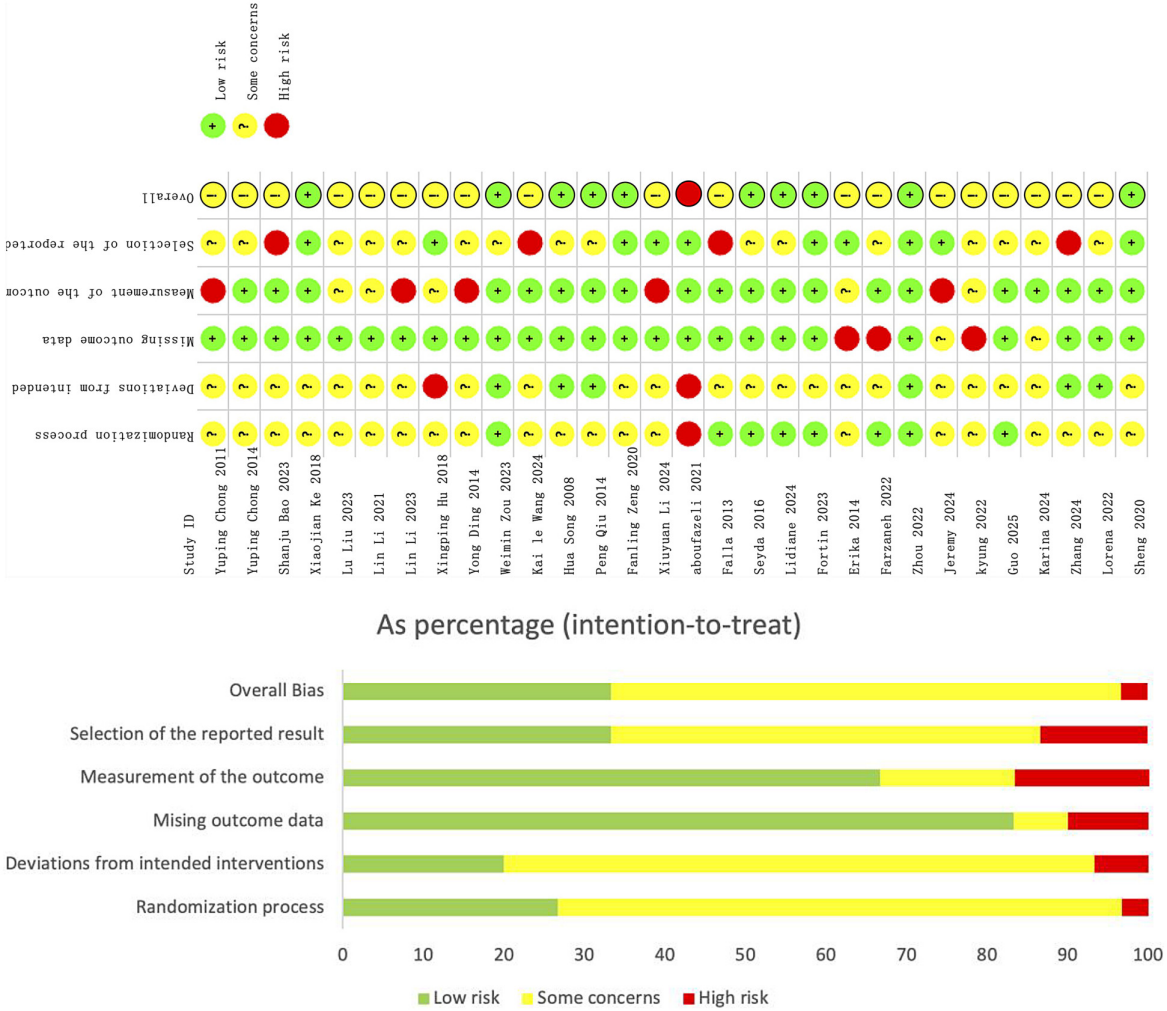
Risk of bias analysis of included literature.

The included literature was evaluated methodologically using the Cochrane 2.0 (44) risk bias assessment tool, referring to Chapter 8 of the 2024 edition of the Cochrane Handbook of Systematic Reviews of Interventions. As shown in Figure 2, 10 studies in the included literature were of high methodological quality, 19 studies had some risk, and 1 study was high risk. Of the total 150 evaluated items, there were 69 low-risk items, 66 intermediate-risk items, and 15 high-risk items, of which 1 study had risk of randomization, 2 studies deviated from established interventions, 3 studies had data on outcome measures that could not be extracted (other outcome measures data were extractable), 4 studies had risk on outcome measures, and 4 studies had risk of publication bias. Subgroup analyses will be used in subsequent analyses to examine whether high-risk studies will have an impact on effect size.

### 3.4 Meta analytical results

#### 3.4.1 VAS pain score

**Figure 3:**
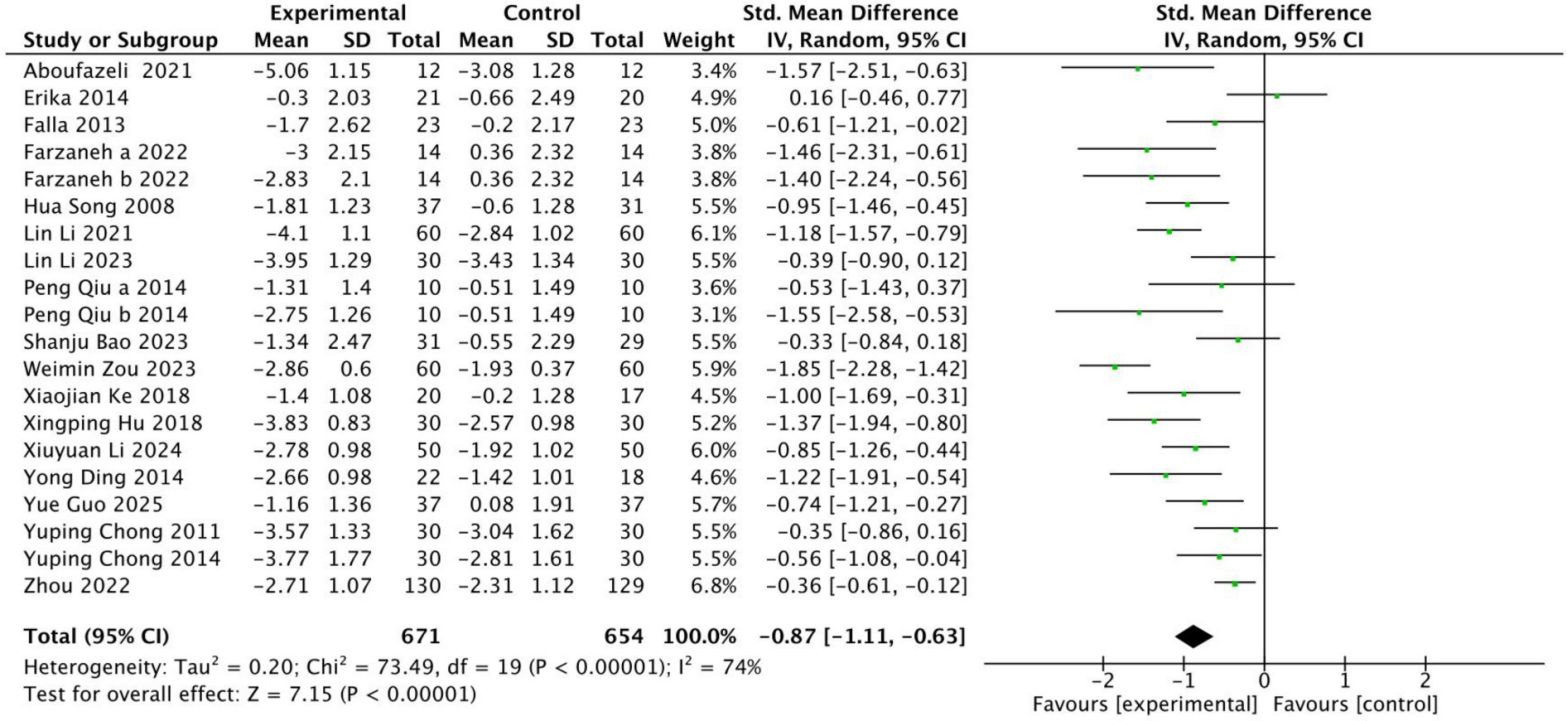
Forest diagram of the impact of different intervention methods on patients' VAS.

**Figure 4:**
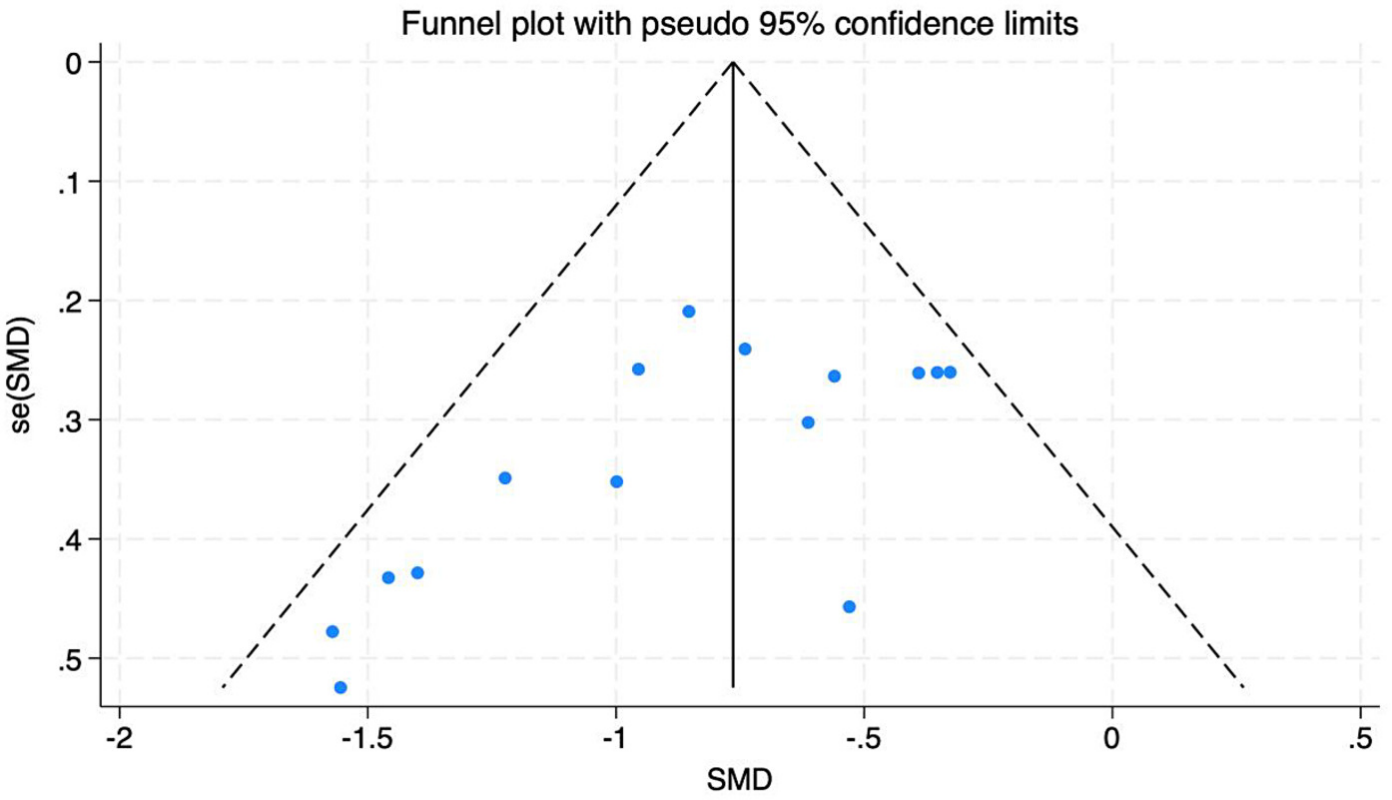
Funnel diagram of the effect of different intervention methods on patient VAS.

A total of 18 articles, containing 20 RCTs, were included to analyze the effects of multiple exercise methods on spinal pain intensity VAS, including 1,325 subjects, 671 in the exercise intervention group and 654 in the control group. There was significant heterogeneity among the studies, with *I*^2^ = 74% data integration using random effects model, as shown in Figure 3. The results of the analysis showed that exercise therapy was superior to the control group in relieving the pain intensity of patients, and the difference was statistically significant (SMD = −0.87; 95% CI: −1.11, −0.63; *P* < 0.05). Through the one-by-one elimination method, it was found that the five studies of Xingping Hu, Erika, Lin Li, Weimin Zou, and Zhou had publication bias, suggesting that these four studies may be the source of heterogeneity. After excluding these four studies, the heterogeneity was significantly reduced, *I*^2^ = 34%. After careful reading of the excluded literature, it was found that in Hu's study, the subjects mainly practiced by themselves through instructional videos, and the intervention effect was poor. In Erika's study, subjects exercised at home and lacked effective supervision, resulting in insignificant curative effect. In Zou's study, the type of disease is neck and shoulder syndrome, which is different from the types of diseases studied in other studies and may be the source of heterogeneity. In Lin Li's study, the subjects in the experimental group had different intervention cycles, larger age span and different intervention cycles, which may be the source of heterogeneity. Zhou's study included a larger number of subjects and higher contribution weight, so it had a greater impact on the overall heterogeneity, as shown in Figure 4. There is an obvious missing angle in the lower right corner of the funnel plot, suggesting that there may be publication bias.

Subgroup analysis was performed by different intervention sites, intervention methods, intervention time, intervention frequency and intervention period and age (Table 2). The results of subgroup analysis showed that in terms of intervention sites, both cervical and lumbar vertebrae improved on VAS, with the most significant improvement in cervical vertebrae (SMD = −0.90); In terms of intervention methods, Zongjianji 18 styles can achieve higher effect size (SMD = −0.98), followed by Baduanjin (SMD = −0.96); In terms of intervention time, the effect size of each intervention was the highest in 10–25 min (SMD = −0.99); In terms of intervention frequency, the effect size of 3–4 times per week (SMD = −1.12) is higher than that of 5–6 times per week (SMD = −1.00); In terms of intervention period, the 20-week intervention has the best effect (SMD = −1.45), followed by 12-week intervention (SMD = −1.17); In terms of age, the elderly group (>60 years) had the highest intervention effect size (SMD = −1.31).

**Table 2:** Subgroup analysis of the impact of different intervention methods on patient VAS.

Covariates	Stratified subgroups	Effect size	95% CI	*P*-value	*I*^2^ heterogeneity
Site of intervention	Cervical cervical	−0.90	(−1.23, −0.58)	<0.01	73%
	Lumbar spine	−0.69	(−1.09, −0.30)	<0.01	83%
	Overall	−0.79	(−1.06, −0.52)	<0.01	81%
Mode of intervention	Yizong Jianji Eighteen Styles	−0.98	(−1.54, −0.42)	<0.01	75%
	Baduanjin	−0.96	(−1.27, −0.65)	<0.01	0%
	Tai Ji Chuan	−0.51	(−0.87, −0.16)	<0.01	56%
	Yoga	−0.46	(−0.82, −0.09)	0.01	0%
	Pulling motion	−0.63	(−1.12, −0.14)	0.01	66%
	Overall	−0.72	(−0.93, −0.51)	<0.01	63%
Time of intervention	10–25 min	−0.99	(−1.36, −0.61)	<0.01	50%
	26–40 min	−0.77	(−1.23, −0.30)	<0.01	58%
	41–60 min	−0.66	(−1.07, −0.24)	<0.01	87%
	Overall	−0.77	(−1.04, −0.50)	<0.01	81%
Frequency of intervention	Once every other day	−0.46	(−0.82, −0.09)	0.01	0%
	1 to 2 times a day	−0.83	(−1.26, −0.41)	<0.01	66%
	1–2 times a week	−0.30	(−1.00, 0.40)	0.40	81%
	3–4 times a week	−1.12	(−1.78, −0.47)	<0.01	89%
	5–6 times a week	−1.00	(−1.26, −0.74)	<0.01	0%
	Overall	−0.80	(−1.07, −0.53)	<0.01	81%
Intervention Cycle	6 weeks	−0.82	(−1.27, −0.37)	<0.01	66%
	8 weeks	−0.75	(−1.07, −0.42)	<0.01	18%
	10 weeks	−0.41	(−1.54, 0.72)	0.48	83%
	12 weeks	−1.17	(−1.64, −0.70)	<0.01	79%
	20 weeks	−1.45	(−2.16, −0.75)	<0.01	–
	24 weeks	−0.95	(−1.46, −0.45)	<0.01	–
	Overall	−0.93	(−1.18, −0.68)	<0.01	74%
Age	<19 years old	−1.13	(−1.49, −0.77)	<0.01	36.3%
	19–44 years old	−0.21	(−0.40, −0.01)	<0.01	55.4%
	44–60 years old	−0.74	(−0.89, −0.58)	<0.01	86.3%
	>60 years old	−1.31	(−1.75, −0.87)	<0.01	0%
	Overall	−0.64	(−0.75, −0.53)	<0.01	84.1%

“–” means not reported.

#### 3.4.2 NDI score

**Figure 5:**
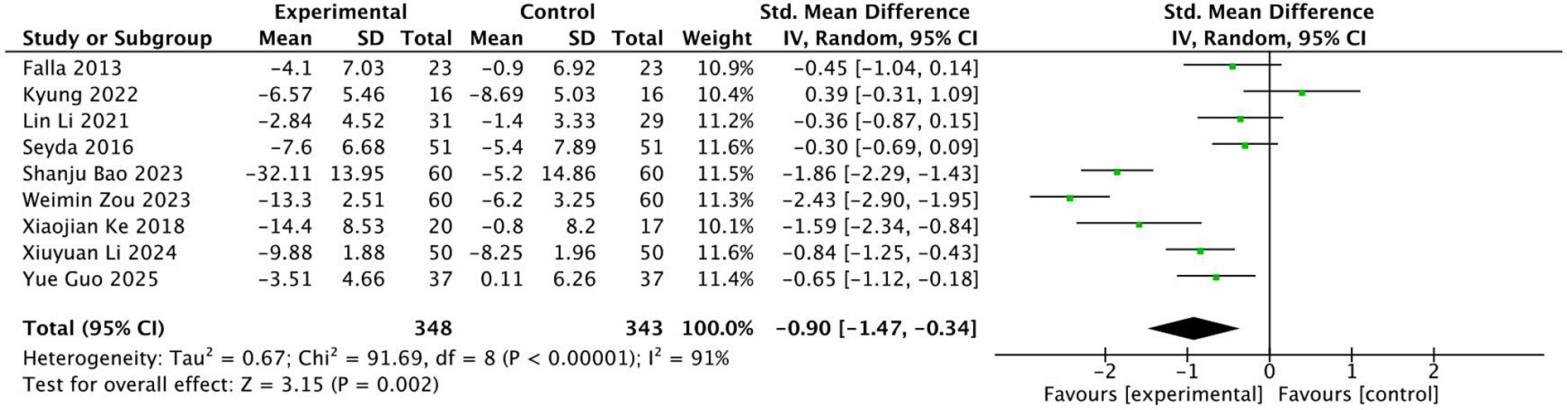
Forest diagram of the effect of different intervention methods on patients' NDI.

**Figure 6:**
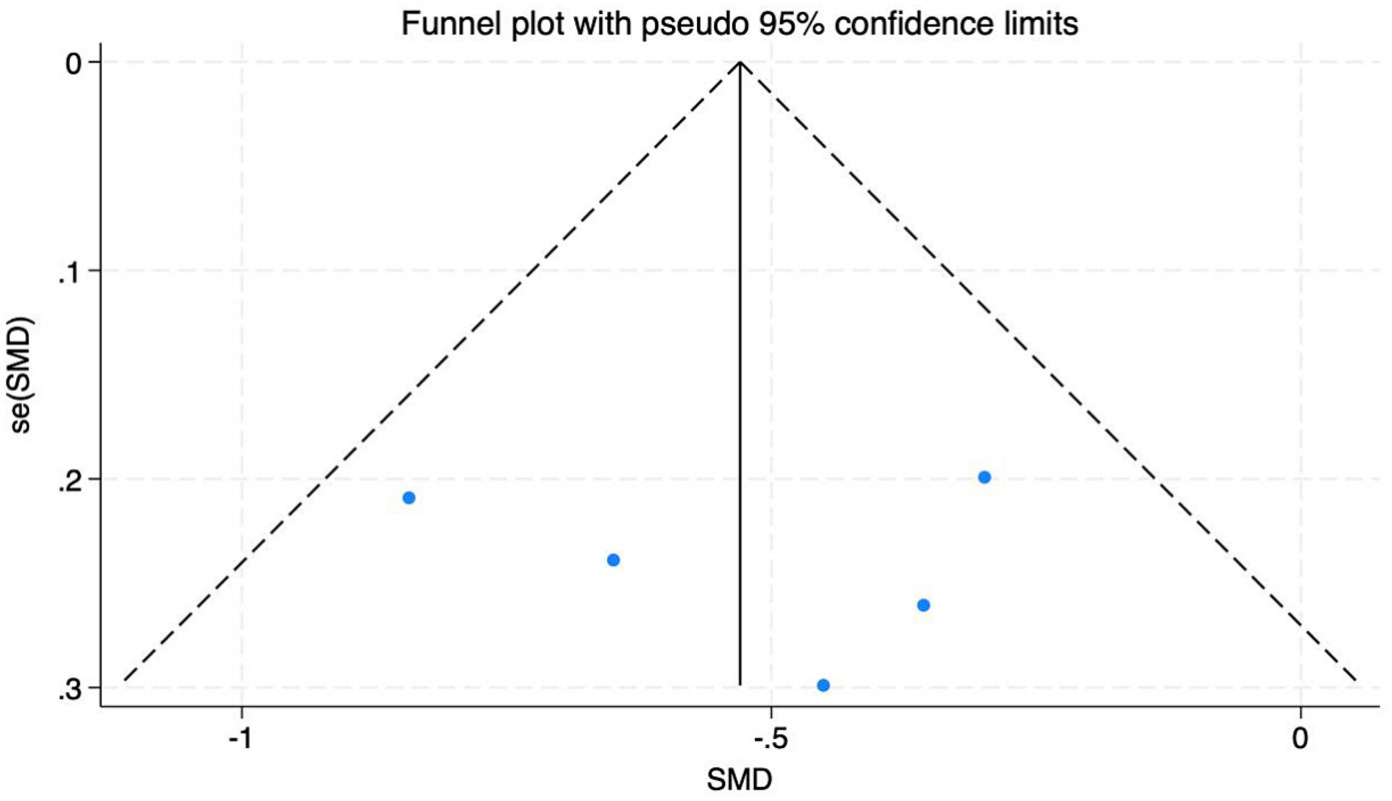
Funnel diagram of the impact of different intervention methods on patient NDI.

A total of nine studies were included with 691 subjects. The heterogeneity test found that there was high heterogeneity among the studies, *I*^2^ = 91%. Therefore, the random effects model is adopted, see Figure 5. The results showed that the NDI of patients in the experimental group was better than that in the control group after exercise therapy, and the difference was statistically significant (SMD = −0.90, 95% CI: −1.47, −0.34, *P* < 0.01). The robustness of the research results and heterogeneous sources were evaluated by eliminating the literature one by one. After eliminating the literature one by one, the overall Meta-analysis results remained robust, indicating that the results were robust to a certain extent. After excluding 4 articles, the heterogeneity was significantly reduced, *I*^2^ = 8%. Because the number of included studies is less than 10, according to the requirements of Cochrane Manual, the funnel plot symmetry test cannot be performed. Here, the funnel plot and Egger test are only used as auxiliary judgment to explore the source of heterogeneity, as shown in Figure 6 and Table 3.

**Table 3:** Egger test of the effect of different intervention methods on patients' NDI.

Std _ Eff	Coefficient	Std.err.	*t*	*p* > *t*	[90% Conf. Interval]
Slope	−1.412651	1.492872	−0.95	0.376	−4.942731, 2.117431
Bias	1.908309	5.970551	0.32	0.759	−12.2098, 16.02642

Subgroup analysis was performed according to different intervention methods, intervention time, intervention frequency and intervention period, as shown in Table 4. The results of subgroup analysis showed that among the intervention methods, Zongjianji Eighteen Forms could achieve a higher effect size (SMD = −1.86), followed by Baduanjin (SMD = −1.15); In the intervention time, the use of 20–30 min intervention can produce a higher effect size, which is statistically significant (SMD = −1.41), while the intervention of 31–50 min and 60 min and above is not statistically significant (*P* > 0.05); In terms of intervention frequency, the effect size of exercise intervention 1–2 times a day was higher (SMD = −1.35), followed by the intervention 5 times a week (SMD = −0.77). In terms of intervention period, the effect size of exercise intervention for ten weeks was the highest (SMD = −1.59), and the exercise intervention lasting for 4–6 weeks was not statistically significant (*P* > 0.05). Because there are few included literatures, no significant heterogeneous sources were found here. Reading the original text found that.

**Table 4:** Subgroup analysis of the impact of different intervention modalities on patient NDI.

Covariates	Stratified subgroups	Effect size	*95% CI*	*P-v*alue	*I^2^* heterogeneity
Mode of intervention	Yizong Jianji Eighteen Styles	−1.86	(−2.29, −1.43)	<0.01	–
	Baduanjin	−1.15	(−1.86, −0.43)	<0.01	66%
	Resistance exercise	−1.03	(−3.80, 1.74)	0.47	68%
	Pulling motion	−0.57	(−0.94, −0.21)	<0.01	0%
	Overall	−1.07	(−1.74, −0.40)	<0.01	63%
Time of intervention	20–30 min	−1.41	(−2.12, −0.71)	<0.01	83%
	31–50 min	−0.32	(−0.71, 0.07)	0.11	50%
	60 min And above	−1.36	(−3.45, 0.73)	0.20	98%
	Overall	−0.90	(−1.47, −0.34)	<0.01	81%
Frequency of intervention	1–2 times a day	−1.35	(−2.34, −0.35)	<0.01	91%
	Once a week	−0.45	(−1.04, 0.14)	0.40	–
	3 times a week	−0.81	(−2.49, 0.87)	0.35	96%
	5 times a week	−0.77	(−1.40, −0.14)	0.02	78%
	Overall	−0.90	(−1.47, −0.34)	<0.01	91%
Intervention Cycle	4–6 weeks	−0.02	(−0.68, 0.65)	0.96	65%
	8 weeks	−0.57	(−0.94, −0.21)	<0.01	0%
	10 weeks	−1.59	(−2.34, −0.84)	0.01	–
	12 weeks	−0.62	(−1.10, −0.15)	0.05	53%
	Overall	−0.53	(−0.88, −0.19)	<0.01	68%

“–” means not reported.

#### 3.4.3 ODI score

**Figure 7:**
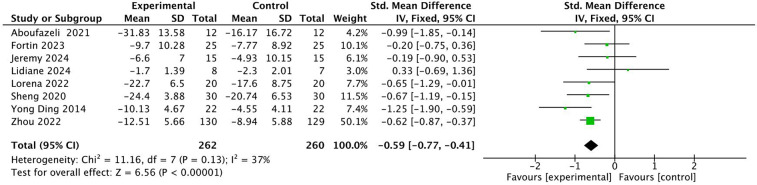
Forest diagram of the effect of different intervention methods on patients' ODI.

**Figure 8:**
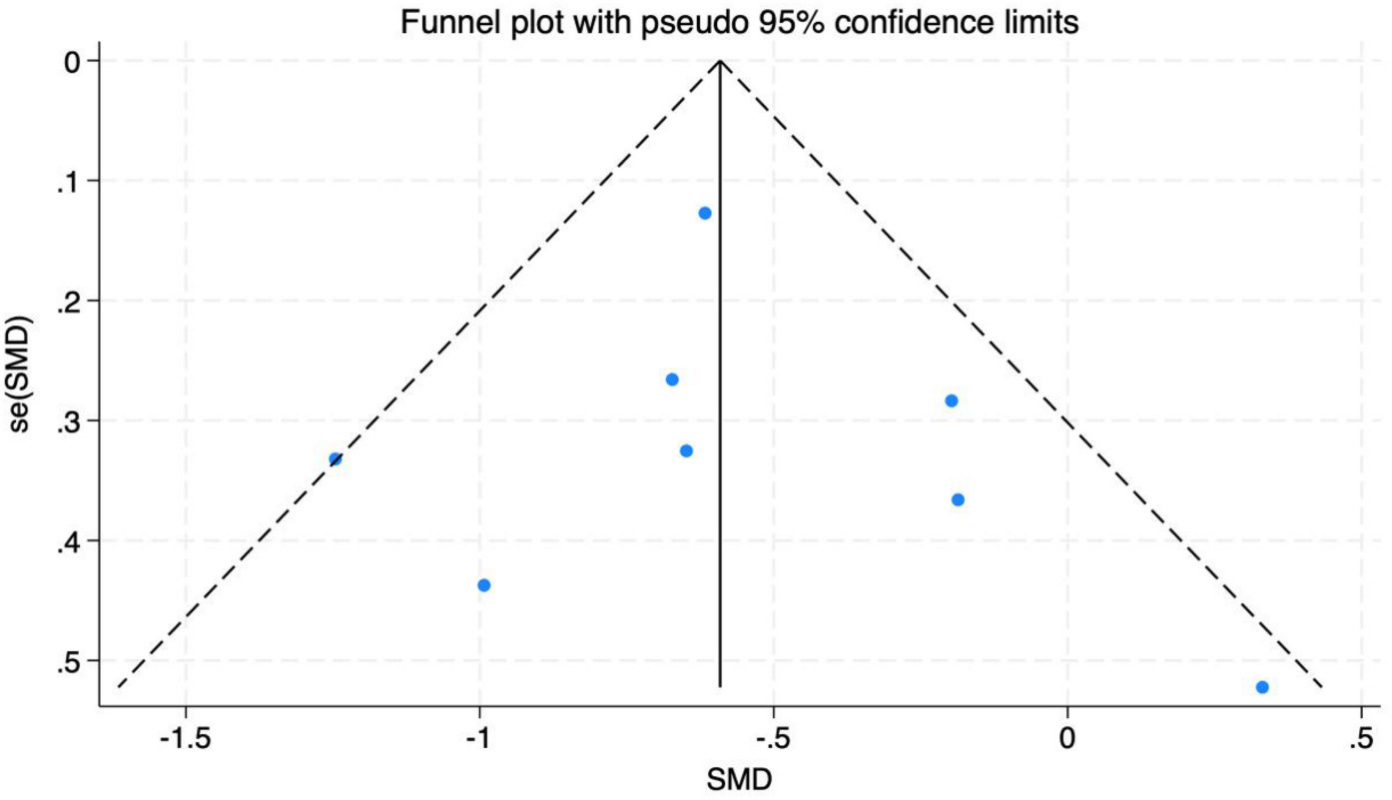
Funnel diagram of the impact of different intervention methods on patients' ODI.

A total of nine studies were included with 652 subjects. The heterogeneity test found that there was low heterogeneity among the studies, *I*^2^ = 31%. Therefore, a fixed-effects model was adopted (Figure 7). The results showed that the ODI of patients in the experimental group was lower than that in the control group after exercise therapy, and the difference was statistically significant (SMD = −0.56, 95% CI: −0.72, −0.41, *P* < 0.01). Through sensitivity analysis and one-by-one elimination method, after excluding one literature, the heterogeneity was significantly reduced, *I*^2^ = 2%. There were less than 10 studies. Here, a funnel plot auxiliary test for publication bias was performed. The funnel plot showed that most studies were uniformly symmetrically distributed, indicating that the degree of bias of the included studies was low, see Figure 8.

Subgroup analysis was performed according to different intervention methods, intervention time, intervention frequency and intervention period (Table 5). The results of subgroup analysis showed that in terms of intervention methods, Baduanjin had the most significant effect (SMD = −0.90), followed by massage (SMD = −0.65), and finally resistance exercise (SMD = −0.53). Pilates and traction exercise were not statistically significant for ODI (*P* > 0.05); In terms of intervention time, the effect of each exercise for 31–50 min was better (SMD = −0.58), while the effect of exercise for 20–30 min was not statistically significant (*P* > 0.05); In terms of intervention frequency, the effect of 5 times a week was the most significant (SMD = −1.25), on the contrary, the frequency of 5 times a day was not statistically significant (*P* > 0.05); In terms of intervention period, the effect of the intervention with 5–6 weeks was more significant (SMD = −0.62), followed by 8–12 weeks (SMD = −0.58).

**Table 5:** Subgroup analysis of the impact of different intervention modalities on patients' ODI.

Covariates	Stratified subgroups	Effect size	*95% CI*	*P-v*alue	*I^2^ het*erogeneity
Mode of intervention	Baduanjin	−0.90	(−1.30, −0.49)	<0.01	45%
	Pilates	0.33	(−0.69, 1.36)	0.53	–
	Massage	−0.65	(−1.29, −0.01)	0.05	–
	Pulling motion	−0.19	(−0.63, 0.25)	0.39	0%
	Resistance exercise	−0.53	(−0.85, −0.21)	<0.01	23%
	Overall	−0.72	(−0.93, −0.51)	<0.01	38%
Time of intervention	20–30 min	−0.44	(−0.96, 0.03)	0.07	0%
	31–50 min	−0.58	(−0.79, −0.37)	<0.01	67%
	60 min	−0.45	(−0.80, −0.11)	0.01	–
	Overall	−0.54	(−0.70, −0.37)	<0.01	42%
Frequency of intervention	5 times daily	−0.19	(−0.90, 0.53)	0.61	–
	1–2 times a week	−0.43	(−0.72, −0.14)	<0.01	23%
	3 times a week	−0.65	(−0.89, −0.41)	<0.01	0%
	5 times a week	−1.25	(−1.90, −0.59)	<0.01	–
	Overall	−0.59	(−0.76, −0.41)	<0.01	38%
Intervention Cycle	5–6 weeks	−0.62	(−0.85, −0.39)	<0.01	0%
	8–12 weeks	−0.58	(−0.94, −0.23)	<0.01	69%
	20 weeks	−0.45	(−0.80, −0.11)	0.01	–
	Overall	−0.57	(−0.74, −0.40)	<0.01	42%

“–” means not reported.

#### 3.4.4 Cobb angle

**Figure 9:**
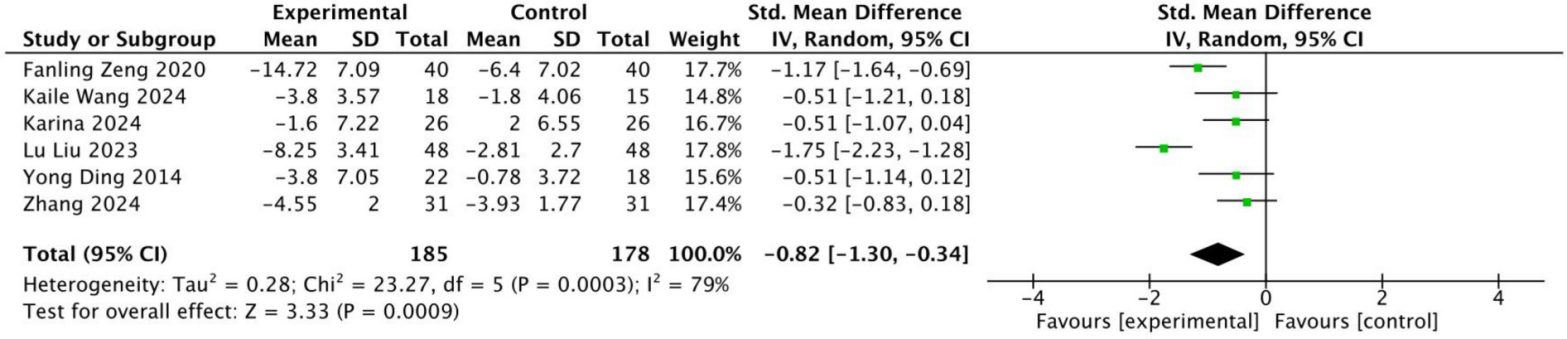
Forest diagram of the effect of different intervention methods on patients' cobb angle.

**Figure 10:**
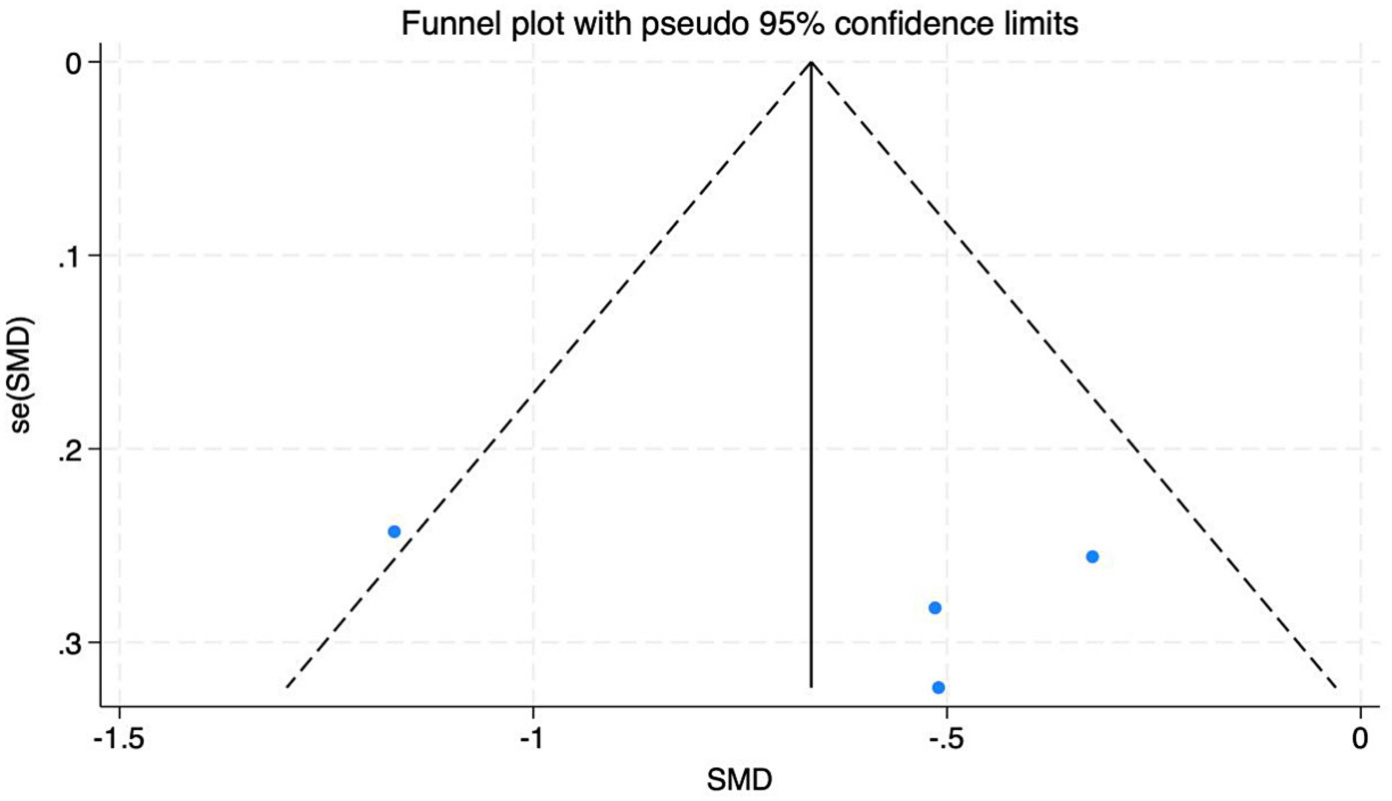
Funnel diagram of the effect of different intervention methods on patient cobb angle.

A total of 6 articles, containing 6 RCTs, were included to analyze the effects of multiple exercise methods on Cobb's angle, including 363 subjects, 185 in the exercise intervention group and 178 in the control group. There was significant heterogeneity among the studies *I*^2^ = 79%, and the random effects model was used for data integration (Figure 9). The results of analysis showed that the Cobb angle of patients in the experimental group was lower than that in the control group after exercise intervention, and the difference was statistically significant (SMD = −0.82, 95% CI: −1.30, −0.34, *P* < 0.01). Through sensitivity analysis and one-by-one elimination method, after excluding 2 (18, 27) articles, the heterogeneity was significantly reduced, *I*^2^ = 0%. By reading the original text, it was found that the intervention frequency of the two articles excluded was significantly higher than that of the other studies, which may result in higher efficacy, which is consistent with the trend of 95% CI of the two studies in the forest graph. Funnel plots showed a uniform symmetrical distribution for most studies, indicating a low degree of bias in the included studies. See Figure 10.

Subgroup analysis was performed according to different intervention methods, intervention time, intervention frequency and intervention period (Table 6). The results of subgroup analysis showed that in terms of intervention methods, Baduanjin had the most significant effect (SMD = −1.02), followed by Schroeter exercise (SMD = −0.41), and core training had no statistical significance for ODI (*P* > 0.05); In terms of intervention time, the effect produced by each exercise for 30–35 min was better (SMD = −1.46), followed by the exercise effect of 40–50 min (SMD = −0.51), while the exercise for 60 min and above was not statistically significant (*P* > 0.05); In terms of intervention frequency, the effect of 1–2 times a day was the most significant (SMD = 1.46), and the frequency of intervention three times a week was not statistically significant (*P* > 0.05); In terms of intervention period, the effect of the 10-week intervention was more significant (SMD = −0.96).

**Table 6:** Subgroup analysis of the effect of different intervention modalities on patient Cobb angle.

Covariates	Stratified subgroups	Effect size	*95% CI*	*P-v*alue	*I^2^ het*erogeneity
Mode of intervention	Schrotter	−0.41	(−0.78, −0.04)	0.03	0%
	Core Training	−0.51	(−1.21, 0.18)	0.15	–
	Baduanjin	−1.02	(−1.68, −0.35)	<0.01	–
	Overall	−0.55	(−0.84, −0.25)	0.05	0%
Time of intervention	30–35 min	−1.46	(−2.04, −0.89)	<0.01	66%
	40–50 min	−0.51	(−0.98, −0.04)	0.03	0%
	60 min And above	−0.32	(−0.83, 0.18)	0.20	–
	Overall	−0.87	(−1.44, −0.31)	<0.01	81%
Frequency of intervention	1–2 times a day	−1.46	(−2.04, −0.89)	<0.01	66%
	3 times a week	−0.39	(−0.80, 0.02)	0.06	0%
	5 times a week	−0.51	(−1.14, 0.12)	0.11	–
	Overall	−0.87	(−1.44, −0.31)	<0.01	81%
Intervention Cycle	10 weeks	−0.96	(−1.35, −0.57)	<0.01	57%
	12 weeks	−0.40	(−0.79, −0.00)	0.05	0%
	2 years	−0.51	(−1.07, 0.04)	0.01	–
	Overall	−0.64	(−0.89, −0.40)	<0.01	41%

“–” means not reported.

## 4 Discussion

### 4.1 Analysis of GRADE evidence quality grading results

As shown in Table 7, the three outcome indicators of VAS, NDI, and Cobb in the GRADE evidence classification are all of moderate quality, and the ODI outcome indicators are of high quality, which improves the credibility of exercise intervention to improve the pain degree, dysfunction, and Cobb angle evidence of patients with spinal health. The main reasons for the moderate quality rating are that some of the included studies describe blinding method, assign hidden situation, and the high heterogeneity in the three outcome indicators of VAS, NDI, and Cobb angle. Therefore, in future research, experimenters should standardize the experimental design and rigorously implement the process, so as to provide more high-quality research for subsequent exercise intervention research on spinal health.

**Table 7:** GRADE evidence level evaluation form.

Outcome measures	Number of documents	Evidence quality evaluation					Sample size		Effect size	Quality of evidence
		Risk of bias^a^	Inconsistency^b^	Indirectness	Imprecision	Publication bias^a^	Exercise intervention	Routine interventions		
VAS	20	seriousness	seriousness	Not serious	Not serious	seriousness	671	654	SMD = −0.87; 95% CI (−1.11, −0.63)	Intermediate level
NDI	9	seriousness	seriousness	Not serious	Not serious	Not serious	348	343	SMD = −0.90, 95% CI (−1.47, −0.34)	Intermediate level
ODI	8	seriousness	Not serious	Not serious	Not serious	Not serious	262	260	(SMD = −0.56, 95% CI (−0.72, −0.41)	Advanced
Cobb	6	seriousness	seriousness	Not serious	Not serious	Not serious	185	178	SMD = −0.82, 95% CI (−1.30, −0.34)	Intermediate level

^a^ Included studies did not describe blinding, assignment concealment.

^b^ Heterogeneity greater than 50%.

### 4.2 Effects of exercise intervention on spinal health

#### 4.2.1 Effect of exercise intervention on VAS

Studies have proved that Visual Analogue Scale (VAS), as a concise and efficient subjective symptom quantification tool, is widely used in the description and measurement of sensory and emotional states due to its unique advantages in multi-time dynamic monitoring and multi-group difference assessment (45). In clinical research, this method is widely used in clinical efficacy evaluation with pain as the primary endpoint because of its high sensitivity to pain perception changes and high reliability of measurement results (46). It is recognized as an effective method to assess pain (47), and it is also one of the commonly used indicators to assess the pain degree of patients with spinal pain. In this study, VAS was used as an outcome indicator to test the effect of different exercise intervention methods on the degree of spinal pain in patients. The 19 articles included included a total of 1,194 patients. The results of the study showed that exercise intervention could significantly improve the degree of pain in patients (SMD = −0.87), which is basically consistent with previous research findings. Zou Weimin et al. (21) used exercise prescription to intervene college students with neck and shoulder syndrome. The results show that exercise prescription intervention can reduce pain by improving the neck and shoulder muscle strength of patients with neck and shoulder dysfunction. Lin Li et al. (16) intervened in patients with cervical spondylosis through Zongjianji 18-style rehabilitation, and the results showed that Zongjianji 18-style rehabilitation exercise could effectively reduce the neck pain of patients. Since then, Lin Li et al. (17) have conducted a study on the intervention of patients with lumbar disc herniation with Zongjianji 18-style combined with dietary nursing. The results show that personalized dietary nursing and Zongjianji 18-style can effectively promote blood circulation and remove blood stasis, reduce inflammatory reaction and relieve the pain of patients. Petersen et al. (48) showed that exercise can induce the secretion of interleukin−6 (IL-6), which can be produced by pro-inflammatory factors such as TNF-α, and at the same time promote the release of anti-inflammatory factors such as interleukin-1ra and interleukin-10. In addition, IL-6 can further inhibit the inflammatory response by activating the AMPK pathway and regulating epinephrine levels. The physiological mechanism of exercise to relieve patients' pain may be that during exercise, the muscle strength around the spine is improved, thus providing stronger support for the tissues around the cervical spine, spine and lumbar spine. At the same time, the repeated stretching and relaxation of muscles during exercise relieves the muscle tension and stiffness caused by long-term bad posture, and achieves the effects of restoring muscle elasticity, improving blood perfusion in painful parts and diluting inflammatory factors in painful parts, thus effectively relieving patients' pain.

The results of subgroup analysis showed that compared with other exercise doses, the best effect size was achieved by VAS intervention with Zongjianji 18-style and Baduanjin exercise intervention for 20 weeks, 3–4 times a week, 10–25 min each time.The reason may be that the 20-week intervention has enough time window to promote muscle remodeling and nerve adaptation. The moderate frequency of 3–4 times a week can not only maintain the anti-inflammatory effect, but also avoid oxidative stress caused by excessive exercise (45). Short-term exercise of 10–25 min each time can improve patient compliance, especially suitable for sedentary people, and ensure long-term persistence in exercise.

#### 4.2.2 Effect of exercise intervention on NDI

Neck-shoulder syndrome is a common cervical function problem, and its pathogenesis is usually related to abnormal pathological changes of cervical spine. The common causes are: muscle injury, nerve root compression and muscle spasm. At the same time, improper posture, cold and humid, and aging may also induce the risk of neck and shoulder syndrome. The cervical spine dysfunction scale (NDI) is modified and compiled according to the Oswestry low back pain index. It is mainly used to evaluate the cervical spine dysfunction in patients with neck pain and acute whiplash, mainly from the common symptoms and functional conditions of cervical spondylosis, and is suitable for many types of cervical spondylosis (49). Studies have shown that the scale has good reliability and validity (50), and can be used to evaluate the functional status of patients with cervical spondylosis (51). In this study, NDI was used as an outcome indicator to evaluate the impact of different exercise interventions on patients' neck pain. The results of Meta-analysis showed that exercise can significantly improve patients' cervical spine dysfunction. This conclusion is effectively confirmed by previous research results. Qiu Peng (24) and others found that Swiss ball instability training can promote the stability of neck excitator muscles and phase muscles, stimulate more muscles to participate in the exercise process, effectively improve the flexibility of shoulder and neck muscle joints, and reduce the symptoms of shoulder and neck dysfunction. Ke Xiaojian (12) and others believe that the movements of “opening the bow left and right like shooting a condor” and “shaking the head and tail to remove the heart fire” in Baduanjin practice have a good effect on the development of neck and back muscles, can effectively increase the stability of cervical spine, and play an important role in the prevention and treatment of neck and shoulder syndrome. Falls et al. (28) found that after 8 weeks of exercise intervention, the NDI score of the training group decreased significantly, indicating that exercise can improve the pain and mobility of patients with chronic neck pain. The mechanism may be due to the significantly improved specificity of neck muscle activity. Exercise enhances the targeted activation of muscles to specific directional forces and reduces the excessive participation of antagonist muscles. In addition, after 8 weeks of exercise intervention, the muscle electromyography of the control group decreased significantly at the sub-maximal contraction, suggesting that muscle coordination and work efficiency were improved. After Guo et al. passed 8 weeks of cervical and thoracic spine “guidance” training, the NDI score of the intervention group decreased significantly. The mechanism of exercise intervention to improve patients' NDI score may be due to the fact that exercise effectively improves the strength of muscles around the cervical spine, stimulates more muscles to participate in the exercise process, antagonist muscles are fully relaxed during training, and neuromuscular flexibility is effectively improved.

Subgroup analysis showed that the best effect size could be achieved by exercise intervention for 10 weeks, 1–2 times a day, 20–30 min each time.

#### 4.2.3 Effect of exercise intervention on ODI

Low back pain is a common disease that seriously affects the quality of patients, which can lead to motor dysfunction and even loss of self-care ability. The etiology of the disease is complex and diverse, mainly including mechanical, chemical and social psychological factors, with pain in the lower back and lumbosacral region as the typical manifestation. Patients are often accompanied by symptoms such as waist weakness, stiffness, limited movement, and decreased coordination, and some patients may have sleep disorders. Pain features are characterized by relief after rest and worsening after bending over, sitting or standing for a long time (52). Oswestry (53) Dysfunction Index (ODI) is a reliable index to evaluate low back pain, and can be used as a reference index to evaluate whether patients with low back pain need of surgery or rehabilitation curative effect. In this study, ODI was used as an outcome indicator to evaluate the effect of different exercise intervention methods on patients' lumbar pain. The 8 articles included included 462 patients, and the overall combined effect size was SMD = −0.55. Exercise intervention can significantly improve patients' lumbar pain, which is consistent with previous studies. Li Huagui (54) and others have shown that the incidence of chronic low back pain is related to lumbar lordosis and sacral inclination angle. Troyanvich (55) showed that excessively lordotic lumbar spine in patients with chronic low back pain can trigger increased back muscle activity, pelvic anteversion and increased sacral inclination angle. Ding Yong (20) and other studies have shown that exercise therapy can effectively enhance patients' lumbar muscle strength, relieve pain and improve lumbar flexibility. Song Hua (23) and other studies mentioned that the twenty-four-style Tai Ji Chuan requires “the tail vertebrae are upright”, that is, the sacrum and caudal vertebrae should be kept upright and not skewed. In addition, alternating muscle contraction and relaxation during practice can effectively promote blood circulation, improve blood perfusion of back muscles, promote the discharge of back metabolites and dilute pain-causing substances. Subgroup analysis showed that Baduanjin exercise intervention for 5–6 weeks, 5 times a week, 31–50 min each time, could achieve the best effect size.

#### 4.2.4 Effect of exercise intervention on Cobb angle

The pathogenesis of scoliosis is complicated, and the specific cause is not yet clear, but the biomechanical characteristics of spinal musculoskeletal play an important role in the progression of scoliosis. In this study, Cobb angle was used as an outcome index to test the influence of different exercise interventions on scoliosis in patients. Cobb angle, defined as the angle between the extension line of the upper end plate of the most inclined vertebral body and the extension line of the lower end plate of the most inclined vertebral body in the curved segment, was adopted by the Scoliosis Research Society (SRS) as a standard method for quantifying scoliosis deformity in 1966 (3). To date, it remains the most commonly used method for assessing spinal curvature. The results of this study showed that a total of 316 subjects were included in 6 articles, and the overall combined effect size SMD was-1.02. Exercise intervention could significantly correct scoliosis in patients. This conclusion is effectively confirmed by previous research results. The guidelines issued by The Society on Scoliosis Orthopaedic and Rehabilitation Treatment (SOSORT) point out that exercise therapy can treat mild AIS (56). Fabio et al. (57) think that exercise can enhance proprioception and motor control of the spine, and will not cause further scoliosis of the spine, which is the best way to treat early scoliosis. Wang Kaile et al. showed the metabolic differences between the two groups of patients before and after training through infrared thermal imaging, indicating that core training can increase muscle work, effectively improve the Cobb angle of patients and prevent pyramidal deformity.

Subgroup analysis showed that the best effect size could be achieved by training of Baduanjin 1–2 times a week for 30–35 min for 10 weeks. In Karina's study (40), the two-year intervention may be affected by factors such as patients' decreased compliance with home exercise, insufficient completion of supervised training, insufficient sample size, and high sample loss, and the intervention effect is poor.

### 4.3 Selection of the best exercise mode and dose

In terms of intervention measures, based on the results of Meta-analysis and subgroup analysis, Zongjianji 18-style and Baduanjin have the best effect size. Among them, Zongjianji 18-style has strong universality, and patients with various spinal diseases can relieve spinal pain by participating in Zongjianji 18-style, while Baduanjin has better curative effect on patients with low back pain and scoliosis.

In recent years, the Eighteen Styles of Zongjianji, as a chiropractic exercise combining traditional medicine and modern science, have been proved to have a significant effect on improving spinal function and relieving neck and low back pain. Based on the chiropractic theory of traditional Chinese medicine, this exercise method combines traditional guidance with modern anatomical physiology and biomechanical research. Through eighteen groups of targeted movements, the related muscles of the spine are systematically exercised to restore the balance of vertebral curvature. Taking Zong Jianji's 18-style style with standardized movement design as the core, the muscles and ligaments around the spine are stimulated through specific postures and movement trajectories. It is considered that the exercise method works through the following ways: (1) By targeted exercise of anterolateral cervical muscles (such as scalene muscle and sternocleidomastoid muscle) and posterior cervical compound muscles (such as head-neck clip muscle and nuchal ligament), the mechanical balance of cervical spine can be restored, the blood supply of vertebral artery can be improved, and symptoms such as dizziness and headache can be relieved; (2) Strengthen the pulling effect of rhomboid muscle and trapezius muscle on thoracic spine, correct thoracic scoliosis, improve thoracic costal joint disorder, and then regulate sympathetic nerve function and relieve autonomic nervous symptoms such as chest tightness and palpitations by holding the shoulders and turning the chest, closing the scapular blades; (3) Enhance the synergistic effect of psoas major muscle and abdominal muscle, restore the physiological curvature of lumbar spine, reduce intervertebral disc pressure, improve spinal canal volume and prevent lumbar spinal stenosis by nodding and bowing, arch forward and arrow back; (4) Improve spinal stability and flexibility, enhance neuromuscular recruitment ability, reduce the proportion of fast muscle fibers, and improve aerobic endurance by simulating daily life movement trajectories, such as cutting steps and turning basin and sitting on bed; (5) Improve the function of internal organs by adjusting intra-abdominal pressure and breathing rhythm, such as increasing the range of thoracic motion by standing up to the sky and promoting the health of respiratory and circulatory system. In addition, this exercise method has significant advantages in time efficiency. The full set of movements only takes 20 min, and the intensity can be adjusted according to individual differences to enhance exercise compliance. It is suitable for daily exercise of sedentary people such as office workers.

In the intervention process of patients with low back pain, Baduanjin has the best intervention effect. As a traditional sports event, Baduanjin has the characteristics of “soft and slow, round and coherent; combination of elasticity and tightness, both movement and static; combination of spirit and form, and integration of qi in it” (58), and its role in the field of spinal health has received more and more attention in recent years. Due to its characteristics of low intensity, slow pace and easy mastery, Baduanjin is considered a rehabilitation and health exercise suitable for people of all ages. The mechanism may be due to the fact that Baduanjin can improve spinal flexibility and enhance the stability of core muscle groups (59). In addition, Baduanjin as a physical and mental integration exercise mode, long-term Baduanjin exercise not only has a rehabilitative effect on spine-related diseases, but also improves overall body functions, such as improving immunity, promoting blood circulation, and enhancing muscle strength (60). Li Hongwei (61) and other studies have proved that Baduanjin can not only relax and exercise the muscles of the waist and back, make the blood circulate better in the waist and back, enhance the stability of the lumbar spine, improve the movement ability of the lumbar spine, and relieve the lumbar pain of patients, but also dredge meridians and strengthen bones, which is conducive to the physical recovery of patients.

Combined with previous studies, PSSE therapy is the first choice for intervention of adolescent scoliosis (8). PSSE is a special training for scoliosis based on biomechanics and exercise rehabilitation principles, combined with three-dimensional posture correction, breathing training and core muscle activation. It is suitable for mild to moderate scoliosis (Cobb angle 10°–40°), especially for adolescent idiopathic scoliosis (AIS). Its schools mainly include: Schroter technique (62), SEAS (scientific exercises approach to scoliosis) (63), BSPTS (Barcelona scoliosis physical therapy school) (64), Dobomed Therapy (65), FITS functional individual training (66), Side Shift therapy, Lyon therapy, breathing control training, joint loosening manipulation, etc. The main training contents include drafting and correction, targeted gymnastics, breathing training, core muscle strengthening, daily posture management, which can give priority to improving Cobb angle and trunk rotation angle; Relieve pain and enhance spinal stability; Delay the progression of scoliosis and reduce the need for surgery; Improve quality of life and correct abnormal breathing patterns. Chang Ying (9) and other studies found that PSSE therapy can significantly reduce the Cobb angle of patients. Compared with other treatment methods, PSSE therapy pays more attention to holistic posture training, emphasizing active three-dimensional self-correction of AIS patients, so that patients can consciously adjust their posture and improve the stability of the spine during correction. In addition, PSSE therapy was more effective in patients with mild AIS (<25°) (67).

In terms of intervention frequency and intervention period, exercise intervention for 10–20 weeks, 2–3 times a week can effectively improve the spinal health of patients. First of all, considering that young and middle-aged patients have less leisure time, elderly patients should not exercise too much, and exercise intervention should be short-term and long-term. In clinical practice, clinicians or rehabilitation therapists can flexibly formulate the best exercise intervention cycle and intervention frequency according to the specific situation of patients and the above recommended scheme.

In terms of intervention time, from the results of subgroup analysis, the intervention time of about 30 min has the best effect. As the intervention time increases, the intervention effect does not even increase but decreases. Long-term exercise easily leads to the fatigue of the sympathetic nerves and parasympathetic nerves of the body, which weakens the central adaptation effect and leads to the reduction of the intervention effect. In addition, most patients are sedentary people, and their basic exercise level is not high. Excessive exercise will harm patients' health. According to the comprehensive results, it is considered that the exercise intervention of about 30 min is the most practical, and it does not affect the patient's study, work and life. In the early stage of exercise, we should pay attention to reasonably controlling the exercise intervention time to prevent excessive exercise.

## 5 Conclusion

Exercise intervention has a significant effect on improving spinal health, which can effectively relieve pain, improve dysfunction and correct scoliosis. Exercise therapy was significantly better than the control group in pain relief (VAS score, SMD = −0.87); In terms of improving dysfunction, cervical dysfunction (NDI score, SMD = −0.90) and lumbar dysfunction (ODI score, SMD = −0.59) have obvious effects; Correction of scoliosis (Cobb angle, SMD = −0.82) is also outstanding.

There are differences in the effects of different exercise modes. Zongjianji Eighteen Poses and Baduanjin show higher effect sizes. The results of subgroup analysis showed that the exercise program of 10–30 min, 3–4 times a week, and continuous exercise for 10–20 weeks could achieve better results.

On the whole, exercise intervention is an effective means to improve spinal health. In clinical practice, doctors or rehabilitation therapists can flexibly formulate personalized exercise intervention plans for patients according to their age, physical condition, leisure time and other specific conditions to achieve the best rehabilitation effect.

## 6 Research limitations and prospects

This study mainly explores the impact of different exercise interventions on patients' spinal health and the best exercise plan. Although the study strictly follows PRISMA guidelines, there are still certain limitations and shortcomings:

(1) Only Chinese and English literatures were searched and included, and there is a possibility that existing related studies are not comprehensively and carefully searched and included.
(2) The exercise modes included in the literature are not concentrated enough, and some exercise modes are less studied.
(3) There were fewer studies that included some of the disorders in the study, and it was impossible to complete a subgroup analysis of the disorder type.
(4) The Cochrane manual points out that when the sample size is less than 10, funnel plot detection cannot be performed. Many outcome indicators in this paper are less included in the literature, and the funnel plot test efficiency is low. The funnel plot test is only used as an auxiliary judgment basis.
(5) Considering factors such as few interventions included in the literature and insufficient sample size, there may be a risk of bias.
(6) The intervention protocol with the highest effect size derived from subgroup analysis requires further validation by mesh Meta-analysis.

In the future, it is hoped that more randomized controlled trials on exercise intervention on spinal health will be included. Therapists and doctors need to be more rigorous in experimental design, and use appropriate randomization methods and blinding methods to reduce the implementation bias, selection bias, publication bias and measurement bias of the study. In the future, it is hoped that there will be enough research to further classify different exercise modes and diseases for in-depth mesh Meta-analysis, so as to explore the best exercise modes and doses for more accurate and comprehensive intervention in different spinal diseases.

## Data Availability

The original contributions presented in the study are included in the article/Supplementary Material, further inquiries can be directed to the corresponding author.
